# Risk analysis of carotid stent from a population-based database in Taiwan

**DOI:** 10.1097/MD.0000000000004747

**Published:** 2016-09-02

**Authors:** Chun-An Cheng, Wu-Chien Chien, Chien-Yeh Hsu, Hui-Chen Lin, Hung-Wen Chiu

**Affiliations:** a Graduate Institute of Biomedical Informatics, Taipei Medical University; b Department of Neurology, Tri-Service General Hospital, National Defense Medical Center; c Department of Medical Research, Tri-Service General Hospital, National Defense Medical Center; d School of Public Health, National Defense Medical Center; e Department of Information Management, National Taipei University of Nursing and Health Sciences, Taipei, Taiwan, ROC.

**Keywords:** carotid stent, nomogram, risk estimate, validation

## Abstract

Because stroke is the third leading disease that causes mortality in the world, the prevention of stroke from advanced carotid stenosis is an important issue. The carotid stent (CAS) is a less invasive to treat advanced carotid stenosis, but for high-risk patients it may cause some events after the procedure that reduces the benefit of stroke prevention. Because patients and their families have less information about risk of events after CAS and are easy concerned, this study calculates the individual probability of major adverse cardiovascular events including any stroke, myocardial infarction, or death after procedure.

The analyzed dataset was composed of patients undergoing CAS from the longitudinal National Health Insurance claim database in Taiwan. The validation dataset was composed of patients undergoing CAS from the Tri-Service General Hospital. We excluded patients under 18 years of age. The prediction model was constructed with a multivariable Cox proportional hazard regression and performed with forward stepwise selection. The nomogram construction was based on the multivariable Cox model.

The risk factors were determined as follows: age with a hazard ratio (HR) of 1.027 (95% confidence interval [CI]: 1.002–1.053) for every 1 year older, congestive heart failure with a HR of 2.196 (95% CI: 1.368–3.524), malignant disease with a HR of 1.724 (95% CI: 1.009–2.944), diabetes mellitus with a HR of 1.722 (95% CI: 1.109–2.674), and symptomatic status with a HR of 1.604 (95% CI: 1.027–2.507). The model showed good discrimination with a *P* < 0.001 (concordance index, 0.681; bootstrap corrected, 0.661) in the derivation data. The concordance index of external validation was 0.66 (*P* = 0.048), which indicates acceptable performance.

We developed a nomogram with a visual scale method and prognostic information, and it is easy to use in clinical practice. The integer-base method may support communication between clinicians and patients before CAS to reduce the anxiety about making a treatment decision. However, insofar as older patients with multiple comorbidities are at high risk, the option of an alternative treatment strategy with medical therapy should be suggested. In the future, prospective tests should be performed to validate whether this model helps patients to prevent events.

## Abstract 2

Supplemental Digital Content is available in the text

## 1 Introduction

Stroke is the third leading disease that causes mortality in the world as well as in Taiwan. Ischemic stroke accounts for 80% of total stroke, and of these, 20% involve carotid stenosis.^[1]^ When carotid stenosis is >50%, it will increase 2-fold the risk of ischemic stroke within 3 years.^[2]^ The outcome of carotid stent (CAS) in both the short and long term has been shown not to be inferior to carotid endarterectomy (CEA) in the Stenting and Angioplasty with Protection in Patients at High-Risk for Endarterectomy (SAPPHIRE), thus providing evidence of alterative therapy for severe carotid stenosis.^[3]^ When carotid stenosis is >70% stenosis, indicating a high risk of ischemic stroke, CAS has been an alternative method in recent years for treating high-risk carotid stenosis and is less invasive than CEA. CAS is a revascularization procedure of the carotid artery, but it may also induce the occurrence of major adverse cardiovascular events (MACE) after the procedure in older patients.^[4–6]^ Because patients and their families have less information and are often concerned about the risk of stroke, myocardial infarction, or death after CAS, we used population-based cohort data to evaluate CAS outcome and create a risk model. Before the CAS procedure, clinicians could explain to patients and their families about the individual risk of MACE within several years.

Nomograms have been widely used for disease prognosis in that they are designed to provide simple numerical estimates of the probability of an event.^[7–9]^ Multiple statistically based independent factors are used to build the nomogram algorithm. Each potential risk factor is assigned a point value and merged with the total number of points, with the values being used to construct a model to predict the outcomes for individual patients. A nomogram for stroke prognosis prediction has already been created with logistic regression to predict long-term outcomes after acute ischemic stroke; it is a user-friendly tool and physicians can apply the one-page nomogram to predict the risk of a disease outcomes.^[8]^

In recent years, there have been several risk scores used for short-term outcomes of patients undergoing CAS.^[10,11]^ In one study, there are 8% mortality within 1 year and 15.8% mortality within 2 years of CAS patients in Medicare beneficiaries; this finding is higher than in clinical trials without the selection of high-risk patients.^[12]^ Some models have also been constructed with categorical scores for mortality prediction.^[13,14]^ These have been made clear the risk of CAS in short-term events and mortality before procedure in individual patients, and the appropriate patients need to be selected to reduce MACE.

Here, we attempt to establish an openly available risk tool with simple numerical estimates for patients considering CAS, for the reason that a risk analysis may help them understand if they may benefit from a reduced event rate after undergoing CAS. Clinicians should communicate to the patient pre-procedurally the various revascularization options and the associated benefits and risks of CAS, so as to enable the patients and their families to make a well-informed decision whether or not to receive CAS. This study combines all statistically significant prognostic predictors into a summary measure for the prediction of a CAS patient's outcome. We also validated a prognostic nomogram that uses a patient's data in a single hospital before procedure to improve ability to predict events for that patient after CAS. The aim of this study was therefore to provide a model to predict individual patient risk within 1 year after carotid artery stenting with internal and external validation.

## 2 Materials and methods

### 2.1 Data sources

The Taiwan National Health Insurance (NHI) was implemented in 1995 and currently covers 99% of Taiwan's 23 million citizens; National Health Insurance Research Database (NHIRD) contains the healthcare information of Taiwan.^[15]^ All formed medical units must submit computerized claim data for medical payment. The dataset used in this study came from the longitudinal National Health Insurance claim database (LNHID), which randomizes selected patients from the population. The validation data used patients undergoing CAS from the period of 2004 to 2015 at the Tri-Service General Hospital, being one of tertiary referral hospital in northern Taiwan. We excluded patients under 18 years of age. The data contained patients’ age, sex, comorbidity, symptomatic status, malignant disease, and MACE and event time.

The study was approved by the Ethics Institutional Review Board of the Tri-Service General Hospital.

### 2.2 Design

The derivation dataset used in this study was obtained from the LNHID in Taiwan from the period of 2004 to 2009. We used the inpatient database of the LNHID to find CAS cases from July 2004 to December 2009. The database contains patient identification numbers, admission date, up to 5 diagnostic and procedure codes from the International Classification of Disease, Ninth Revision, Clinical Modification (ICD-9-CM). On the basis of the NHI payment guide, carotid artery stent is to be performed for symptomatic patients with carotid stenosis ≥60% or asymptomatic patients with carotid stenosis ≥80% with a higher risk of cardiopulmonary condition. We searched for patients who received CAS while ICD-9-CM diagnostic code was 433 and ICD-9-CM procedure code was 39.90 or 39.50. We excluded patients under 18 years of age. The first admission time was defined as the index date.

We defined MACE with ischemic stroke with ICD-9-CM codes from 434 to 437 and hemorrhage stroke (430–432) after the index date or after receiving computer tomography (ICD-9-CM procedure codes: 87.03 or 87.04) and magnetic resonance image (ICD-9-CM procedure code: 88.91) at the index date, myocardial infarction with ICD-9-CM from 410, or death after CAS from the death database. Patients were followed from the CAS until the first event or December 31, 2009. Comorbidities found by ICD-9-CM codes included diabetes mellitus (250), hypertension (401–405), atrial fibrillation (427.31), chronic obstructive pulmonary disease (490–492, 494, 496), hyperlipidemia (272), congestive heart failure (428), coronary artery disease (410–414), and malignant disease (140–208), chronic kidney disease (586), end-stage renal disease (585; ICD-9-CM procedure codes: V42.0 indicating renal transplant, 39.95 indicating hemodialysis, 54.97 indicating peritoneal dialysis), we classified chronic kidney disease stage 2 to 4 was chronic kidney disease except end-stage renal disease.

The validation data were retrieved from patients undergoing CAS from 2004 to 2015 at a tertiary medical center in northern Taiwan. We reviewed the patients’ charts and found age, sex, comorbidities, MACE and event time following up within 1 year.

### 2.3 Statistical analysis

Descriptive statistics were computed to summarize the MACE and non-MACE groups of the derivation dataset; numerical variables were tested with Student *t* test; categorical variables were tested with χ^2^ test; and a *P* < 0.05 was taken to indicate significance. The prediction model was constructed with a multivariable Cox proportional hazard regression model and performed with forward stepwise selection, and only statistically significant variables (*P* < 0.05) were retained. The model displays the relationship between predictors and the hazard function of a particular failure time. The statistical computations were performed using SPSS software version 18.

### 2.4 Nomogram

The nomogram was constructed by the rms package in R software.^[16]^ The point scale was assigned points to these variables in the nomogram based on the multivariable Cox proportional regression model. The sum of the points assigned for each variable was rescaled to a range from 0 to 100, and a straight line was drawn upward to determine the points for the variables. The points of the variables were accumulated and marked in the total points. The probabilities of MACE-free survival at 1 year are found by drawing a vertical line from the total points’ axis straight downward to the outcome axes.

### 2.5 Internal validation and external validation

The concordance index provides the ability of the nomogram to correctly discriminate a patient's individual MACE risk. An internal bootstrap validation for 200 times resamples was performed to correct the overfitting bias of testing on the same patient population and to discriminate the free event times of 2 patients (concordance index). Kaplan–Meier curves were plotted to assess the MACE-free survival of patients categorized in the risk group for internal validation. Log-rank tests were used to compare the predictive value averaged over time between patients in the low, intermedia, and high-risk group.^[7]^ The external validation data evaluated the concordance index and was illustrated by Kaplan–Meier curves with log-rank test to analyze the difference of survival curves.^[17]^

## 3 Results

### 3.1 Demographic data

There were 317 patients who received CAS from the in derivation dataset and 137 patients in the validation dataset. The outcome of CAS patients was followed up for a mean time of 2.26 years in derivation dataset. A total of 7 patients suffered short-term MACE, there were 6 (1.9%) ischemic stroke, 1 (0.3%) cerebral hematoma, 5 (1.6%) myocardial infarction and 3 (1%) died within the periprocedural period. Forty-four patients (13.9%) suffered MACE in the derivation dataset, and 26 patients (18.9%) had MACE in validation dataset within 1 year. During the overall study period, 51 patients (16%) died, 36 (11.4%) had cerebral vascular accidents, 21 (6.6%) had myocardial infarction, and a total of 84 patients (26.5%) had MACE after CAS in the derivation dataset (see Figure, Supplement Digital Content, flow chart of derivation and validation dataset of undergoing CAS).

We found that older patients with more comorbidities (72.98 ± 9.72 years compared with 69.64 ± 9.72 years) will experience MACE in the derivation dataset, and that patients in the MACE group had more diabetes mellitus, atrial fibrillation, chronic obstruction pulmonary disease, congestive heart failure, chronic kidney disease, and malignant disease (Table 1). More chronic kidney disease, hyperlipidemia, and symptomatic status were noted in the validation data because we checked the laboratory data and carefully reviewed the chart information. A comparison of the derivation dataset and validation dataset is shown in Table 2.

**Table 1:**
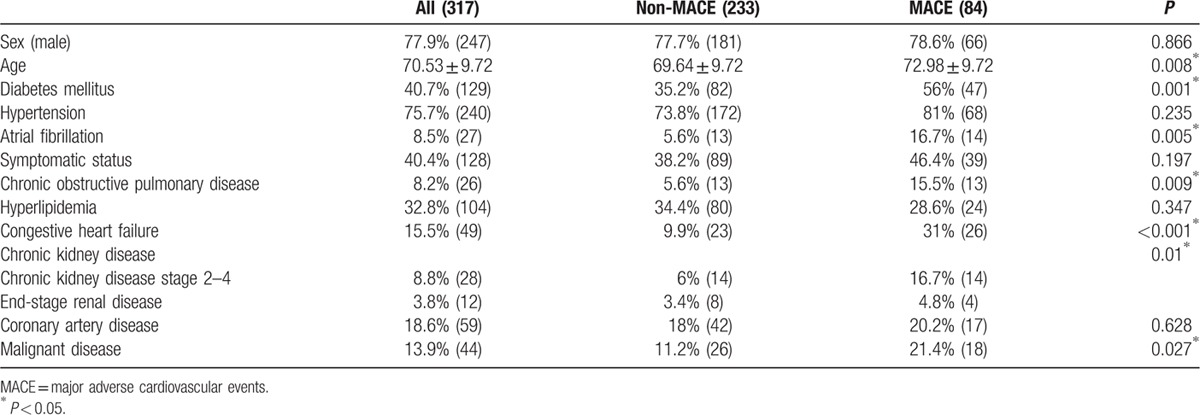
Baseline characteristics in derivation dataset with or without major adverse cardiovascular events.

**Table 2:**
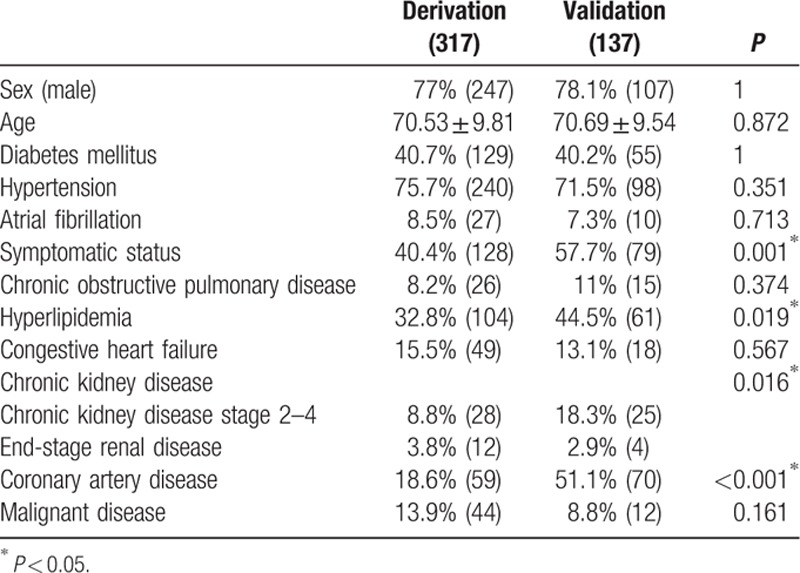
The characteristics of patients in diffident dataset.

The causes of mortality in the derivation dataset were 8 tumors (15.7%), 7 sepsis with infection (13.7%), 7 cerebral infarction (13.7%), 6 coronary artery disease (11.8%), 6 diabetes mellitus (11.8%), 4 congestive heart failure (7.8%), 2 intracerebral hemorrhage (3.9%), 2 ischemic bowel disease (3.9%), 2 chronic obstructive pulmonary disease (3.9%), 2 cardiopulmonary failure (3.9%), 1 acute kidney failure, gastrointestinal bleeding, liver cirrhosis, hypertension, and spondylosis (2%). We analyzed the patients suffering from cerebral vascular attacks and myocardial infarction increased mortality rate by 2.78 (95% CI: 1.236–6.244), and by a 6.465 (95% CI: 2.531–16.51) crude odds ratio rate after CAS.

### 3.2 Risk factors of outcome after carotid stenting

The Cox proportional hazard regression model revealed 5 risk factors after CAS: age, congestive heart failure, symptomatic status, diabetes mellitus, and malignant disease. Congestive heart failure was the strongest risk factor with a hazard ratio (HR) of 2.196 (95% CI: 1.368–3.524). Malignant disease was the secondary risk factor with a HR of 1.724 (95% CI: 1.009–2.944). Diabetes mellitus was a risk factor with a HR of 1.722 (95% CI: 1.109–2.674). Symptomatic CAS patients carried a risk with a HR of 1.604 (95% CI: 1.027–2.507). Age was a risk factor with a HR of 1.027 (95% CI: 1.002–1.053) for every 1 year older.

### 3.3 Nomogram construction

**Figure 1:**
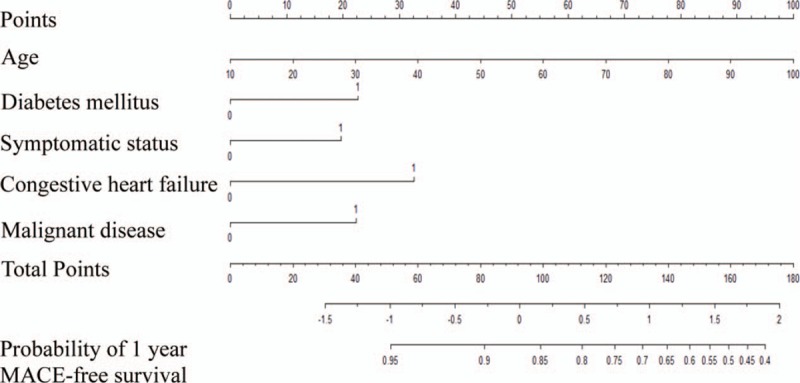
Nomogram for probability of major adverse cardiovascular events undergoing carotid stent. MACE = major adverse cardiovascular events.

**Figure 2:**
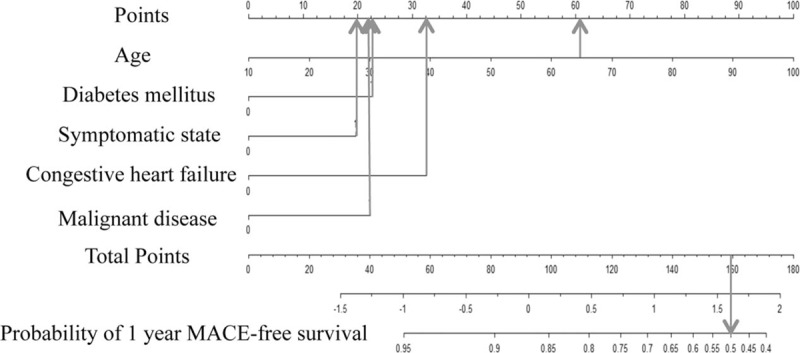
Nomogram application. A straight line was drawn upward to determine the points for the variables (up arrow). For example, a patient aged 65 years (61 points) and with diabetes mellitus (23 points), symptomatic status (20 points), congestive heart failure (33 points), and malignant disease (22 points) receives a total point score of 159. The probabilities of MACE-free survival at 1 year is found by drawing a vertical line from the total points’ axis of 159 straight downward to the outcome axes with of 50% (down arrow) and a 50% probability of MACE. MACE = major adverse cardiovascular events.

The nomogram is a graphical scale that provides the estimated probabilities for outcomes, and the resulting classifications based on age and comorbidity revealed by Cox proportional hazard regression. In this nomogram, the point was contributed to the largest scale according to the age. The other parameters were congestive heart failure (33 points), diabetes mellitus (23 points), malignant disease (22 points), and symptomatic status (20 points) (Fig. 1). For example, a patient aged 65 years (61 points) without comorbidity corresponds to an estimated probability of 93.3% of MACE-free survival in 1 year. In other words, the probability of MACE is 6.7%. Another patient aged 65 years (61 points) with diabetes mellitus (23 points), symptomatic status (20 points), congestive heart failure (33 points), and malignant disease (22 points) has a total point score of 159, which corresponds to an estimated probability of 50% of MACE-free survival in 1 year and a 50% probability of MACE in 1 year (Fig. 2). When 65-year-old patients with the other 4 risk factors receive CAS, they will have a 7.46 times greater occurrence of MACE than those of the same age without the risk factors.

### 3.4 Internal and external validation

**Figure 3:**
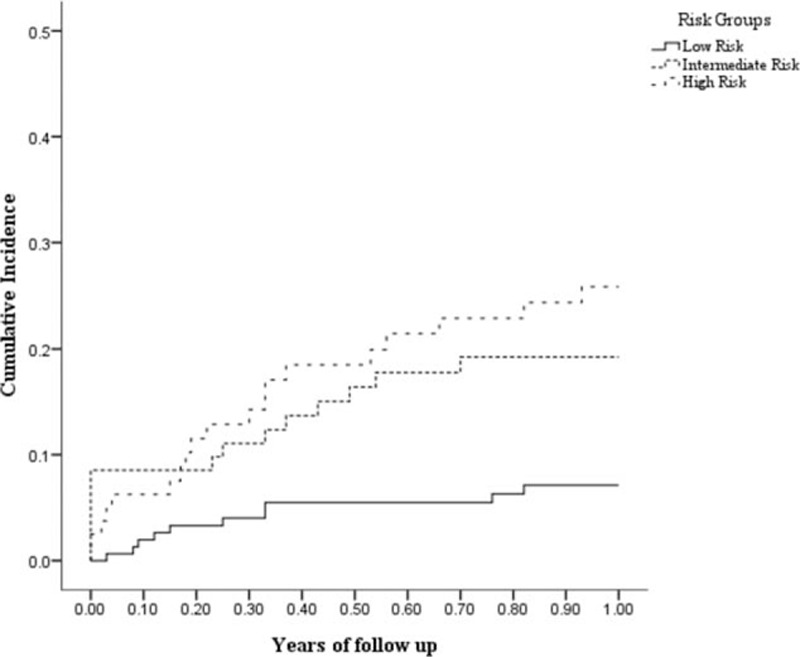
One year cumulative incidence of MACE in NHIRD dataset according to risk groups: 6.5% MACE occurred during low-risk group, 18.3% MACE occurred during intermedia group, and 23.7% MACE occurred during high-risk group (log-rank test: *P* = 0.001).

**Figure 4:**
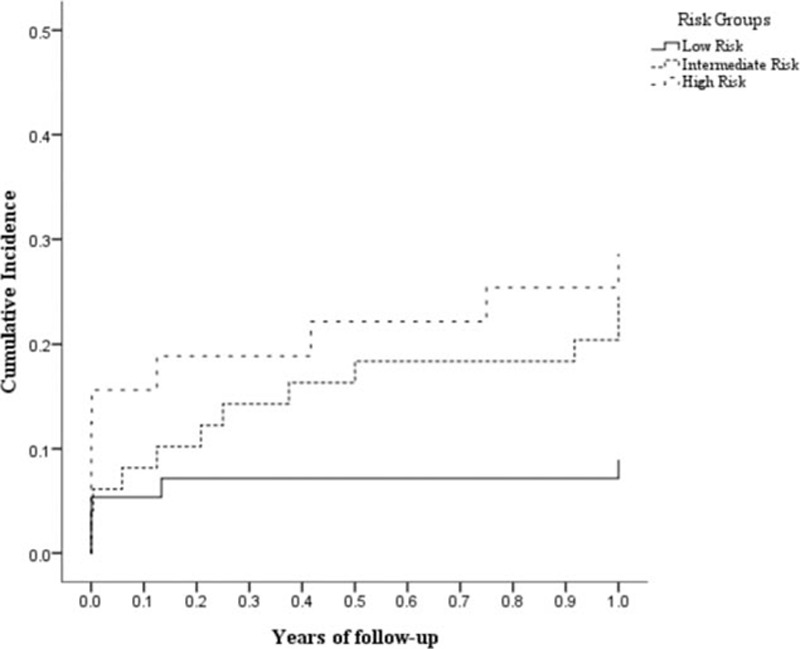
One year cumulative incidence of MACE in validation dataset according to risk groups: 8.9% MACE occurred during low-risk group, 24.5% MACE occurred during intermedia group, and 28.1% MACE occurred during high-risk group (log-rank test: *P* = 0.041).

The model was shown to have good discrimination with a *P* < 0.001 (concordance index, 0.681; bootstrap corrected, 0.661) in the derivation dataset. MACE-free survival time was determined according to risk stratification based on nomogram-predicted MACE-free survival probabilities. The concordance index of external validation was 0.66 (*P* = 0.048). Patients were classified according to the predicted probability of MACE-free survival. The means of the total points were 108.37 ± 28.11 in the MACE group and 90.32 ± 24.37 in the non-MACE group in derivation dataset. We set 3 groups, the first (total points < 90) group was the low-risk group, the second (90 ≤ total points < 108) was the intermediate risk group, and the final (total points ≥ 108) was the high-risk group. The low-risk group (score<90) contained 155 patients (48.9%) with 1 year MACE of 6.5%. The intermediate risk group (90≤score<108) contained 82 patients (25.9%) with 1 year MACE of 18.3%. The high-risk group (score≥108) contains 80 patients (25.2%) with 1 year MACE of 23.7%. Figure 3 illustrates the Kaplan–Meier survival curves according to nomogram-based risk groups, and the cumulative incidence of MACE was significantly differentiated between the groups in the NHIRD dataset (log-rank test: *P* = 0.001). Figure 4 illustrates the Kaplan–Meier survival curves according to nomogram-based risk groups, and the cumulative incidence of MACE was significantly differentiated between the groups in the validation dataset (log-rank test: *P* = 0.041). This optimized risk score discriminated well from low to high risk (*P* < 0.05) in both the internal and external validation. HR across risk groups showed in Table 3; HR of high-risk group versus low-risk group was 3.954 (95% CI: 1.839–8.505, *P* < 0.001) in derivation data; HR of high-risk group versus low-risk group was 3.491 (95% CI: 1.168–10.429, *P* = 0.021); and HR of intermediate risk group versus low-risk group was 2.902 (95% CI: 1.022–8.841, *P* = 0.035) in validation data.

**Table 3:**
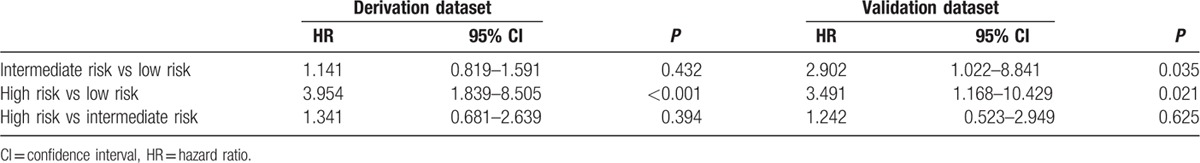
Hazard ratios evaluated in the derivation and validation dataset.

## 4 Discussion

This study identified the prognosticators of long-term events in patients having CAS. After the analysis of risk factors, a predictive scoring system was developed to better estimate the events following the CAS procedure. This long-term risk model can help determine the individual risks of CAS patients and may assist clinicians to provide better risk estimates to patients, rather than simply classifying the risk of patients. Because patients may have high-risk factors that will induce MACE after CAS and reduce the benefit of CAS to prevent stroke due to high mortality and cardiac events, clinicians can better select suitable patients to perform CAS by using this nomogram.

We used the Cox regression model to reveal the risks associated with older age, diabetes mellitus, congestive heart failure, symptomatic status, and malignant disease after undergoing CAS. According to this risk model that evaluates a patient's condition, physicians can identify high-risk patients and discuss the possibility of MACE with patients and their families when considering CAS as a treatment option. We developed a nomogram to predict the probability of MACE after CAS based on clinical information from a population-based database. Although older age and lesion-related factors influence the short-term risk of patients undergoing CAS,^[10,11]^ we found that age and comorbidities may be important for long-term outcomes. On the basis of a statistical selection, age was the most important influence risk factor revealed in this nomogram. Symptomatic status and older age are the 2 most clearly risk factors for CAS in experts’ experience^[18]^ and from Medicare data.^[12]^ Similar to a previous report that symptomatic status is a risk factor for mortality in hospital stroke,^[10]^ we also found a higher frequency of short-term stroke, mortality, and long-term events in symptomatic patients. Congestive heart failure was also observed to affect CAS outcome in a German registry study^[19]^ as well as in a previous efficacy and safety study in Taiwan.^[20]^

CAS is a less invasive procedure to treat advanced carotid stenosis, and it is being increasingly used as a treatment for CAS in place of CEA. However, given that long-term MACE seems to affect as many as one-fourth of the patients, clinicians need to carefully select patients for CAS. Although patients receiving CAS for revascularization have a reduced occurrence of ischemic stroke, there is a high risk of MACE for patients with advanced age and the burden of comorbidities. Because understanding risk is important in decision making, previous groups have developed scores to predict the short-term adverse events for individual patients undergoing CAS,^[10,11]^ and we propose our constructed nomogram for the risk prediction of long-term outcomes. Patients’ condition and anatomic factors were used to develop a model and an integer-based risk score to predict stroke or death within 30 days in the SAPPHIRE worldwide study. Short-term risk prediction scores for patients at high risk for CAS increases with age, clinical comorbidity, carotid artery, and carotid stenosis characteristics.^[11]^ Another study used age, symptomatic status, absence of prior CEA, atrial fibrillation, prior stroke, and impending major surgery to develop a prediction model for in-hospital stroke or death, to identify the risks of individual patients during the admission period.^[10]^ A clinical rule found under 75 years female patients with contralateral stenosis and restenosis having low risk for CAS.^[21]^ Older and symptomatic patients were found in Medicare data to have a higher short- and long-term mortality risk for CAS, and with only limited benefit.^[12]^ A mortality risk model of a previous study indicated that older age >80 years (score 1), diabetes mellitus (score 1), coronary artery intervention (score 2), severe chronic kidney disease defined but not on dialysis (score 2), dialysis patients (score 3), and chronic obstructive pulmonary disease (score 3) influenced the 3-year survival of patients undergoing CAS. The score ≤2 predicted a 6.0% 3-year mortality, whereas score >2 was associated with a 31.6% 3-year mortality.^[14]^ Age of >70 years had more atherosclerosis and comorbidities, with a frequent occurrence of coronary disease or stroke. Patients with congestive heart failure have poor cardiac function and increased embolism formation. Heart failure is also known to increase embolic risk during coronary angiography and cardiac invention, and it is the strongest independent predictor for death or stroke in hospitals from a German registry study.^[20]^ The underlying poor cardiac condition affects the outcome of CAS patients. Patients with diabetes mellitus have a high risk for cardiovascular disease in their lifetime, with diabetes mellitus combining with hyperlipidemia and inflammation processes to cause atherosclerosis.^[22,23]^ Poor blood sugar control leads to an increase of intima thickness, and one study revealed that patients aged 68 years or older and an intima thickness of 1.5 mm are factors that can separate high-risk from low-risk populations.^[24]^

It is doubtful of benefit if patients do not live long enough or too high procedural risk whether performing CAS. Reducing the risk of stroke can be achieved successfully by carotid revascularization after undergoing CAS, but it has been noted that high-risk patients do not survive longer and thus do not benefit from stroke reduction. Owing to the higher mortality rate (16%) in the derivation dataset compared with clinical trial of Carotid Revascularization Endarterectomy Versus Stenting Trial (CREST) study (11.3%), the same finding was observed in Medicare beneficiaries.^[12]^ When patients consider CAS as a means to prevent future strokes, the clinician must explain the benefit only if the patients can have a prolonged life expectancy because the CREST study excluded some patients with dialysis, malignant disease, and the other severe underlying conditions. The decision to receive CAS should be based on consideration of overall survival time and the risk of cardiac complication, and high-risk patients should be recommended to undertake conservative treatment to reduce MACE.

There were more long-term stroke and death risks undergoing CAS than CEA in a systematic review.^[25]^ According to the real-world data, CAS is a relative risky procedure. We used 5 factors to develop a prediction model that we hope will provide an acceptable risk estimate for patients as well as their families and physicians. The NHIRD is a long-term follow-up database, from which we used the retrospective cohort data of patients with CAS and validated the model with a hospital data. It is important to pay attention to the CAS patients’ underlying conditions, and then carefully select relatively low-risk patients to undergo the procedure. Our findings demonstrate higher rate of long-term MACE after undergoing CAS in patients with multiple risk factors, and for this reason low-risk patients should be selected for CAS to reduce MACE probability. The awareness of the individual patient's event risks may have an important impact on the management of the patient, including more frequent clinical visits and a tendency of conservative treatment with medication for high-risk carotid stenosis patients.

There are several limitations in this study. First, carotid artery lesion characteristics have been previously found to influence short-term outcome, but we enrolled data from the NHIRD that does not contain information on lesion characteristics of carotid stenosis and severity of comorbidity. Second, because we could not distinguish advanced carotid stenosis with medical treatment, we did not enroll advanced carotid stenosis without revascularization as a control group to evaluate the relative risk of CAS. Third, the mean following time was 2 years in the derivation dataset and 1 year in the validation data, so a longer follow-up time is needed to evaluate more long-term outcomes in the NHIRD in the future. Fourth, the concordance index of model was statistical significantly, but only fair as 0.661.

## 5 Conclusion

We found that older patients with significant comorbidity undergoing CAS seem to have a higher risk of adverse events. We developed nomogram with visual scale method with reliable prognostic information that is easy to use in clinical practice. We used this nomogram with individualized risk assessment so as to identify high-risk patients and provide more detailed information about the risks to them or their relatives before CAS to support rational decision making. In the future, a prospective test should be performed to validate whether this model helps patients prevent events and promote their understanding.

## Supplementary Material

Supplemental Digital Content
