# Supplementary material for: Risk analysis of carotid stent from a population-based database in Taiwan
Source: Medicine (Baltimore). 2016 Sep 2;95(35):e4747. doi: 10.1097/MD.0000000000004747 (PMC5008606; doi:10.1097/MD.0000000000004747)
Supplement: Supplemental Digital Content [file medi-95-e4747-s001.doc]

MACE

n=26

Non-MACE

n=111

Non-MACE

n=272

MACE

n=45

Non-MACE

n=233

MACE

n=84

1 year period

Overall 2.26 years

1 year period

Excluded

n=1

Age<18

Excluded

n=1

Age<18

Validation dataset

n=137

Derivation dataset

n=317

Carotid stent patient in Tri-Service General Hospital from July 2004 to December 2015

n=138

Carotid stent patients in NHIRD from July 2004 to December 2009

n=318

Supplemental Digital Content. Flow chart of derivation and validation dataset of undergoing carotid stent.

NHIRD: National Health Insurance Research Database
